# A Glycosyl Hydrolase 5 Family Protein Is Essential for Virulence of Necrotrophic Fungi and Can Suppress Plant Immunity

**DOI:** 10.3390/ijms25052693

**Published:** 2024-02-26

**Authors:** Xiaofan Liu, Huihui Zhao, Jiatao Xie, Yanping Fu, Bo Li, Xiao Yu, Tao Chen, Yang Lin, Daohong Jiang, Jiasen Cheng

**Affiliations:** 1 State Key Laboratory of Agricultural Microbiology, Huazhong Agricultural University, Wuhan 430070, China; 2 The Provincial Key Laboratory of Plant Pathology of Hubei Province, College of Plant Science and Technology, Huazhong Agricultural University, Wuhan 430070, China

**Keywords:** *Sclerotinia sclerotiorum*, glycosyl hydrolase, virulence, immune response

## Abstract

Phytopathogenic fungi normally secrete large amounts of CWDEs to enhance infection of plants. In this study, we identified and characterized a secreted glycosyl hydrolase 5 family member in *Sclerotinia sclerotiorum* (SsGH5, *Sclerotinia sclerotiorum* Glycosyl Hydrolase 5). *SsGH5* was significantly upregulated during the early stages of infection. Knocking out *SsGH5* did not affect the growth and acid production of *S. sclerotiorum* but resulted in decreased glucan utilization and significantly reduced virulence. In addition, *Arabidopsis thaliana* expressing SsGH5 became more susceptible to necrotrophic pathogens and basal immune responses were inhibited in these plants. Remarkably, the lost virulence of the *ΔSsGH5* mutants was restored after inoculating onto *SsGH5* transgenic *Arabidopsis*. In summary, these results highlight that *S. sclerotiorum* suppresses the immune responses of *Arabidopsis* through secreting SsGH5, and thus exerts full virulence for successful infection.

## 1. Introduction

*Sclerotinia sclerotiorum* (Lib.) de Bary is a destructive ascomycete fungus with a wide host range [1,2]. This necrotrophic fungus can infect more than 700 species of plants and cause significant yield losses in many crops [3,4]. Equally important, the complexity of the disease cycle and pathogenic mechanism makes this fungus difficult to control [5,6]. Study of the pathogenesis of *S. sclerotiorum* will help to develop environmentally friendly strategies for *Sclerotinia* disease control.

*Sclerotinia sclerotiorum* has long evolved a powerful arsenal to combat plants, and numerous studies have shown that the successful infection of this fungus is closely linked to the production of oxalic acid (OA), cell-wall-degrading enzymes (CWDEs) and effectors [7,8,9]. OA plays important roles in many aspects of the plant immune response, such as providing a low pH environment for invasion, calcium sequestration, impairing the oxidative burst of the plant, induction of programmed cell death (PCD), and inhibition of autophagy [10,11,12,13,14,15,16]. Moreover, small secretory proteins are also involved in the pathogenesis of *S. sclerotiorum*. A large number of secreted proteins are predicted for *S. sclerotiorum* and the functions of some of them have been empirically studied. For example, *S. sclerotiorum*-secreted proteins SsCP1, SsITL1, SsSSVP1, and SsPINE1 interact with different plant important proteins, thereby inhibiting plant immunity and promoting infection [17,18,19,20]. Likewise, importantly, this fungus is induced by the host to secrete large amounts of CWDEs to break down plant cell walls and digest plant cells, ultimately facilitating successful infection [21,22,23,24].

A large number of studies have shown that CWDEs play key roles in the infection of many phytopathogens. For example, pectate lyases enzymes are important virulence factors in the pathogenic fungi *Colletotrichum coccodes* and *Verticillium dahliae* [25,26]. In addition, some CWDEs, showing xylanase and hemicellulase activities are essential for the virulence of *Valsa mali* and *S. sclerotiorum* [27,28]. In some cases, although CWDEs are very important to the virulence, they can also be recognized by host plants. For example, PsXEG1 is a protein of the GH12 family and an important virulence factor in *Phytophthora sojae*. It exerts xyloglucanase activity and recognized as PAMP by the RXEG1-BAK1 protein complex of plants [29,30]. Not coincidentally, a GH17 family protein CfGH17 in tomato pathogen *Cladosporium fulvum* has β-1,3-glucanase activity, and its expression in tomato can cause plant cell death [31]. It has also been reported that xylanases of *V. dahliae* containing a GH11 domain are important for the virulence of *V. dahliae*, and can cause cell death in *Nicotiana benthamiana* leaves [26]. Interestingly, recent studies have shown that CWDEs can help pathogens avoid host plant recognition. For instance, MoChia1 belonging to the GH18 family can not only regulate the growth of *M. oryzae*, but also binds to its own chitin to escape plant immunity during infection [32]. A GH17 extrafamily β-1,3-glucanase also can help *M. oryzae* escape plant recognition by hydrolyzing β-glucan during early infection [33]. These studies show that the mechanism of CWDEs in the process of pathogen infection is diversified. Although *S. sclerotiorum* secretes large amounts of CWDEs during infection, detailed information on the virulence-related functions of CWDEs has been rarely reported, and their role and mechanism in infection need to be further clarified. 

In this study, we identified a secretory CWDE of *S. sclerotiorum*, which contains a GH5 family domain and is significantly upregulated during the early stage of infection. We named it SsGH5 (*Sclerotinia sclerotiorum* Glycosyl Hydrolase 5). By phenotyping *SsGH5* knockout mutants and *SsGH5* transgenic *Arabidopsis*, we found that SsGH5 is essential for the full virulence of *S. sclerotiorum*, and this protein can suppress the immune responses of *Arabidopsis*. This study first confirms the biological function of a core CWDE of *S. sclerotiorum*, which contributes to further understanding the virulence mechanism of *S. sclerotiorum* and thus to developing methods to address plant diseases caused by this destructive pathogen. 

## 2. Results

### 2.1. Secretary Protein SsGH5 Is Conserved in Ascomycetes

**Figure 1:**
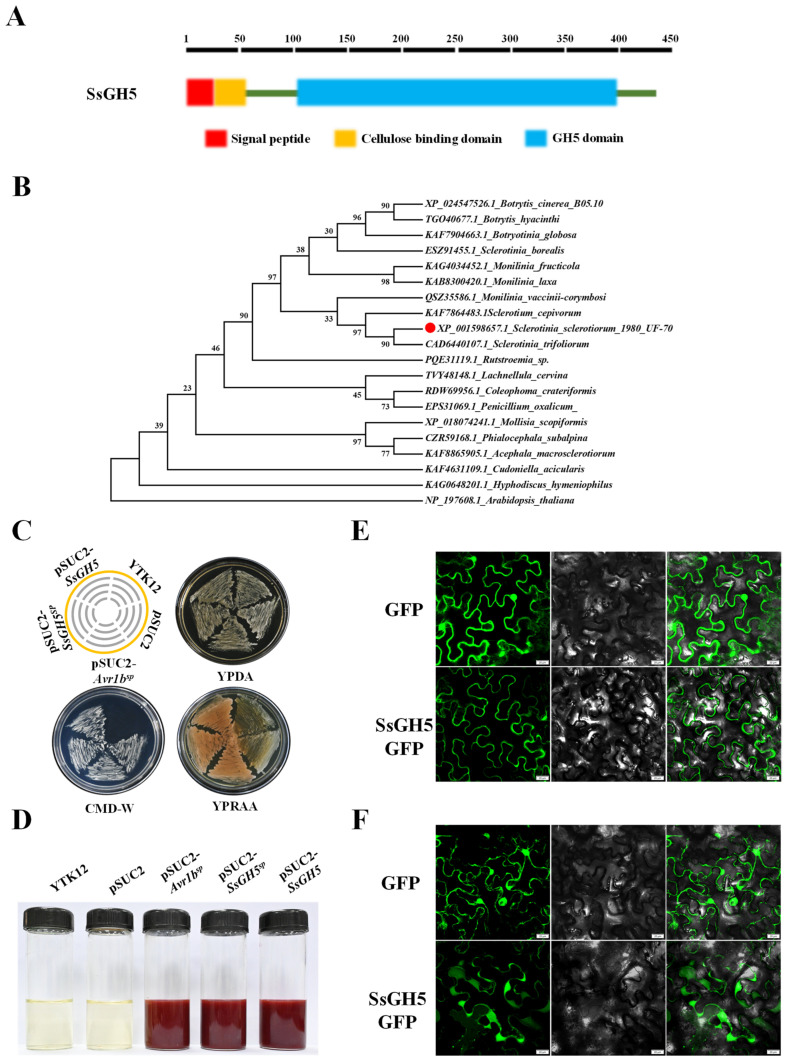
Secretary protein SsGH5 is conserved in *Ascomycetes*. (**A**) SsGH5 (red circle) is a secreted glycosyl hydrolase family 5 protein containing 440 amino acids, including a signal peptide (SP), a cellulose-binding domain and a glycosyl hydrolase family 5 structural domain. (**B**) Phylogenetic relationships of SsGH5 with other fungal homologs were determined by maximum likelihood. Branch length is proportional to the average probability of the change in features on that branch. Phylogenies were constructed using Mega X with the neighbor-joining method. (**C**) Secretory function of the SsGH5 signal peptide was verified by the yeast secretion trap screen assay. The signal peptide and intact protein of SsGH5 were fused in frame with the yeast invertase sequence in a pSUC2 vector and expressed in the YTK12 strain. The functional signal peptide of Avr1b was used as a positive control, while the YTK12 and pSUC2 empty plasmids were used as negative controls. (**D**) Invertase activity in TTC solution. TTC encountered sucrose breakdown products to produce triphenylformazan, which showed a red reaction to confirm that the functional signal peptide enables a sucrose-converting enzyme to be secreted. (**E**) SsGH5 green fluorescent fusion protein, expressed in *N. benthamiana* leaves by the *Agrobacterium* system to observe the subcellular localization of SsGH5, and GFP protein expressed as a control. (**F**) Subcellular localization of SsGH5 green fluorescent fusion protein in *N. benthamiana* leaves under plasmolysis, and GFP protein expressed as a control. The pictures were taken 48 h post-agroinfiltration with confocal laser scanning microscopy. And *N. benthamiana* cells were treated with 1 M NaCl for 3 min to observe plasmolysis. Bars, 20 μm.

Recent studies have shown that the function of GH family proteins in different plant pathogens is very important. Our previous study showed that *SsGH5* expression was significantly upregulated during *S. sclerotiorum* infection [9]. SsGH5 encodes a 440 aa protein, amino acids 1–19 at the N-terminus of SsGH5 are a predicted signal peptide (SP), amino acids 23–50 are a predicted cellulose-binding domain, and residues 104–402 contain a typical glycosyl hydrolase 5 family domain (Figure 1A). Phylogenetic analysis showed that SsGH5 and its direct homologs from different *Ascomycetes* fungi are evolutionarily conserved and that its arginine and lysine are highly conserved among the direct homologs (Figure 1B and Figure S3). To determine that the SsGH5 signaling peptide has normal secretory activity, we performed experimental validation by the yeast secretion trap system [34]. YTK12 with a pSUC2-Avr1b^sp^ vector was used as a positive control, and YTK12 and YTK12 with a pSUC2 vector were used as a negative control (Figure S1a). YTK12 strains transformed with pSUC2-SsGH5^sp^ or pSUC2-SsGH5 vectors grew normally on YPRAA medium and produced insoluble red triphenylformazan when treated with 2, 3, 5 chlorotriphenyltetrazole (TTC) (Figure 1C,D). To clarify the subcellular localization of SsGH5, we expressed a fusion protein of SsGH5-GFP in *N. benthamiana* leaves, and green fluorescence appeared in the periphery of the cells as observed by laser confocal microscopy (Figure 1E). We further treated the *N. benthamiana* leaves with 1 M NaCl to observe plasmolysis, and the fluorescence of SsGH5-GFP expression aggregated in the apoplast of *N. benthamiana* cells (Figure 1F). These results strongly suggested that SsGH5 is a secreted protein conserved in *Ascomycete* fungus.

### 2.2. SsGH5 Is a Glycosyl Hydrolase Specifically Induced by the Host

**Figure 2:**
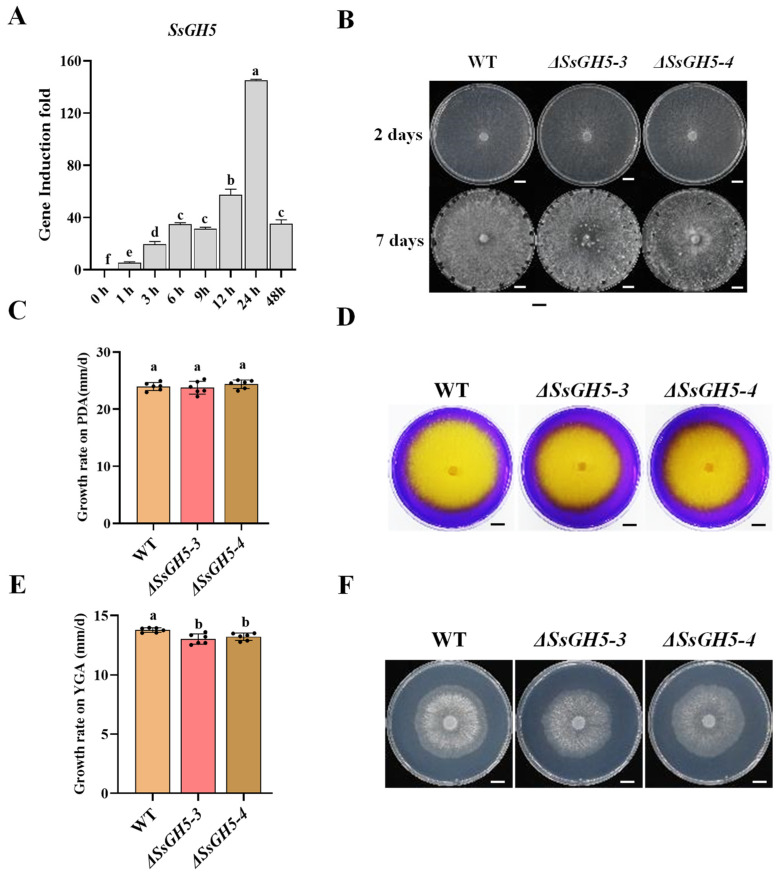
SsGH5 is a glycosyl hydrolase specifically induced by the host at the *S. sclerotiorum* infection stage. (**A**) Relative levels of SsGH5 transcript accumulation were determined by RT-qPCR in inoculated *Arabidopsis* plants. Levels of *β-tubulin* transcripts of *S. sclerotiorum* were used to normalize different samples. Values are similar in three independent experiments. (**B**) Measurement of the wild-type (WT), *ΔSsGH5-3* and *ΔSsGH5-4* strain growth rates utilizing the cross-over method at 20 °C on potato dextrose agar (PDA). Upper graph bar, 1 cm. (**C**) Colony morphology of the WT, *ΔSsGH5-3* and *ΔSsGH5-4* strains grown at 20 °C for 15 days on PDA. (**D**) Acid-producing ability of the WT, *ΔSsGH5-3* and *ΔSsGH5-4* strains was determined on PDA containing 0.005% (*w*/*v*) bromophenol blue dye as an indicator. The yellow color appearing in the medium indicated acid production by mycelia. Upper graph bar, 1 cm. (**E**) Measurement of the WT, *ΔSsGH5-3* and *ΔSsGH5-4* strain growth rates utilizing the cross-over method at 20 °C on YGA. (**F**) Colony morphology of the WT, *ΔSsGH5-3* and *ΔSsGH5-4* strains grown at 20 °C for 2 days on YGA. Upper graph bar, 1 cm. These values are the mean ± S.E. Different letters on the same graph indicate statistical significance at *p* < 0.01 using one-way ANOVA.

To further clarify the expression pattern of *SsGH5*, real-time quantitative PCR (RT-qPCR) was performed. Results indicated that when *S. sclerotiorum* was inoculated onto the leaves of the host, the transcript level of *SsGH5* increased rapidly and reached the peak (145-fold) at 24 h post-inoculation (hpi) (Figure 2A). This result suggested that the transcription of *SsGH5* is specifically induced by the host and this gene may play a significant role during *S. sclerotiorum* infection. To further investigate the function of *SsGH5*, we obtained two *SsGH5* deletion mutants (*ΔSsGH5-3* and *ΔSsGH5-4*) by targeting the replacement gene with a hygromycin resistance cassette and validated them at the DNA and RNA levels (Figure S1b–d). The growth rate, colony morphology, and oxalic acid production capacity of the two *ΔSsGH5* mutants were similar to those of the wild-type strain 1980 (Figure 2B–D), suggesting that *SsGH5* is not required for normal growth and does not affect oxalic acid production. SsGH5 belongs to glycosyl hydrolase family 5, a family of proteins commonly reported to have cellulase activity [35]. Therefore, we hypothesized that SsGH5 may have the ability to hydrolyze cellulose. β-glucan is a type of cellulose and one of the main components of plant cell walls [36]. When the WT and the *SsGH5* knockout mutant strains were cultured on the medium with yeast extract, β-glucan and agar (YGA), the growth rate was lower than that of the WT (Figure 2E), but the colony morphology of these strains was not significantly different (Figure 2F). These results indicated that SsGH5 is a glycosyl hydrolase that is specifically upregulated at the stage of infection.

### 2.3. SsGH5 Is Essential for the Full Virulence of S. sclerotiorum

**Figure 3:**
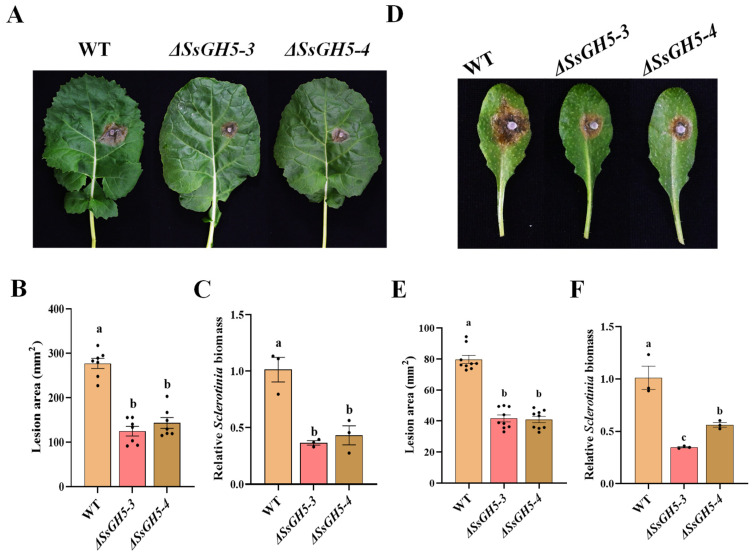
SsGH5 is essential for the full virulence of *S. sclerotiorum*. (**A**) Virulence assay of the *S. sclerotiorum* WT, *ΔSsGH5* mutants *ΔSsGH5-3* and *ΔSsGH5-4* on *B. napus*. The symptoms were photographed at 36 hpi. (**B**) Measurement of lesion area by the cross-over method. (**C**) Equal area samples from infected sites were used to extract DNA, and the relative biomass was analyzed by RT-qPCR. (**D**) Virulence assay of the *S. sclerotiorum* WT, *ΔSsGH5* mutants *ΔSsGH5-3* and *ΔSsGH5-4* on *Arabidopsis* Col-0. (**E**) Measurement of lesion area by the cross-over method. (**F**) Fixed mass samples of susceptible parts were selected and DNA was then extracted for subsequent detection of the amount of pathogen and plant endogenous genes using RT-qPCR, and we then performed relative biomass analyses. These values are the mean ± S.E. Different letters on the same graph indicate statistical significance at *p* < 0.01 using one-way ANOVA.

To clarify the function played by SsGH5 during the infection by *S. sclerotiorum*, we inoculated the WT, *ΔSsGH5-3* and *ΔSsGH5-4* strains onto 45-day-old *Brassica napus* (Westar) leaves, and photographed and counted lesion sizes after 36 h. The results showed that the *ΔSsGH5* knockout strains had a significantly lower lesion area compared to the WT (Figure 3A). The mean lesion area of the WT was 276 mm^2^, while that of the *ΔSsGH5-3* and *ΔSsGH5-4* strains was only 124 mm^2^ and 143 mm^2^, respectively (Figure 3B). And the biomass statistics showed that the *ΔSsGH5* knockout strains also had significantly lower biomass than the WT (Figure 3C). Similarly, virulence assay of these strains on 4-week-old *A. thaliana* (Col-0) leaves showed that the virulence of *SsGH5* knockout strains was significantly reduced compared to that of the WT (Figure 3D–F). In conclusion, these results confirmed the essential role of SsGH5 in promoting full virulence during *S. sclerotiorum* infection.

### 2.4. SsGH5 Inhibits Plant Immunity and Makes Plants More Susceptible to Necrotrophic Fungi

To further investigate the role of SsGH5 in the infection of plants by *S. sclerotiorum*, stable transgenic plants (*35S:SsGH5-3* × *Flag*) constitutively expressing *SsGH5* were generated in wild-type *Arabidopsis* (Col-0). The expression of *SsGH5* in transgenic *Arabidopsis* was confirmed through Western blot (Figure S2a). Compared with WT Col-0, the size of *SsGH5* transgenic lines (*oxSsGH5-3*, *oxSsGH5-6*, *oxSsGH5-14*) is slightly smaller (Figure S2b,c). 

**Figure 4:**
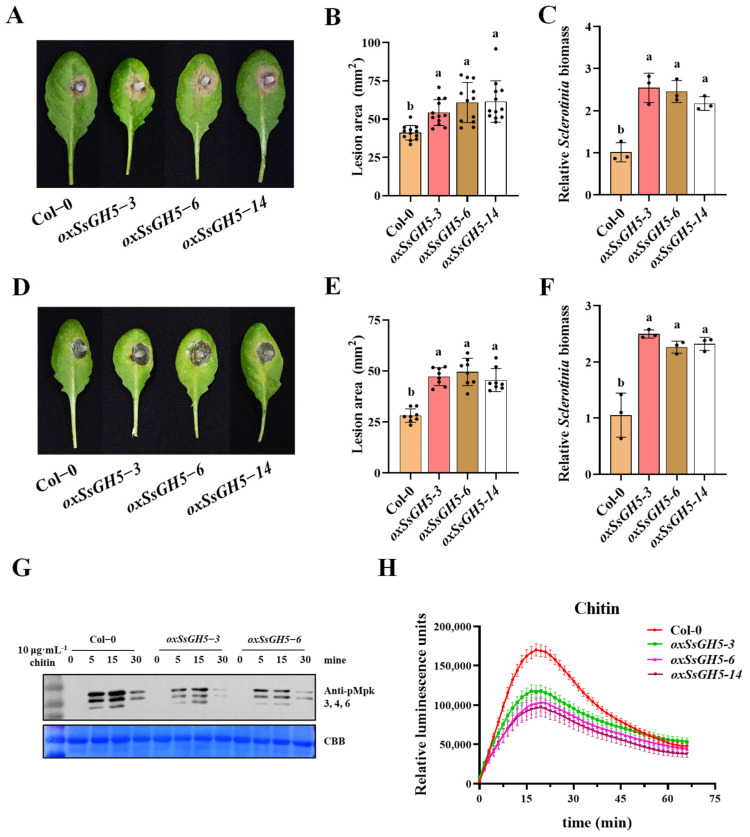
SsGH5 sensitizes plants to necrotrophic fungi and inhibits plant immunity. (**A**) Virulence assay of the *S. sclerotiorum* WT 1980 strain on Col-0 and SsGH5−3 × Flag-expressing (*35S:SsGH5*) lines at 30 hpi. (**B**) Measurement of lesion area by the cross-over method. (**C**) Equal area samples from infected sites were used to extract DNA, and the relative biomass was analyzed by RT-qPCR. (**D**) Virulence assay of the *B. cinerea* WT B05.10 strain on Col-0 and SsGH5−3 × Flag-expressing (*35S:SsGH5*) lines at 36 hpi. (**E**) Measurement of lesion area by the cross-over method. (**F**) Equal area samples from infected sites were used to extract DNA, and the relative biomass was analyzed by RT-qPCR. These values are the mean ± S.E. Different letters on the same graph indicate statistical significance at *p* < 0.01 using one-way ANOVA. (**G**) Expression of SsGH5 inhibited chitin-induced phosphorylation of MAPKs. MAPK activation was detected by immunoblotting with α-PERK antibody (top). Protein loading is shown by Coomassie Brilliant Blue (bottom). (**H**) Detection of chitin-induced ROS burst in Col-0 and SsGH5 transgenic lines. Leaf discs from 4-week-old plants were treated with 10 μg·mL^−1^ chitin and values represent the means ± SE (*n* = 12 biological replicates).

We first inoculated *SsGH5* transgenic plants with *S. sclerotiorum*, and the result showed that transgenic *Arabidopsis* expressing SsGH5 was more susceptible to t *S. sclerotiorum* (Figure 4A), the lesion area of the transgenic *Arabidopsis* increased by 20% to 30% compared with that of Col-0 (Figure 4B), and the relative biomass statistics of lesion area showed consistent trends (Figure 4C). Likewise, the resistance of SsGH5 transgenic plants to *B. cinerea*, another necrotrophic fungal pathogen, was also significantly reduced (Figure 4D–F). In addition, SsGH5 transgenic plants showed a remarkably significant reduction in both chitin-triggered ROS burst and MAPK activation (Figure 4G,H). Taken together, *SsGH5* transgenic *A. thaliana* exhibited significantly diminished immunity and heightened susceptibility to necrotrophic fungi.

### 2.5. SsGH5 Is an Important Pathogenic Factor of S. sclerotiorum Infection

**Figure 5:**
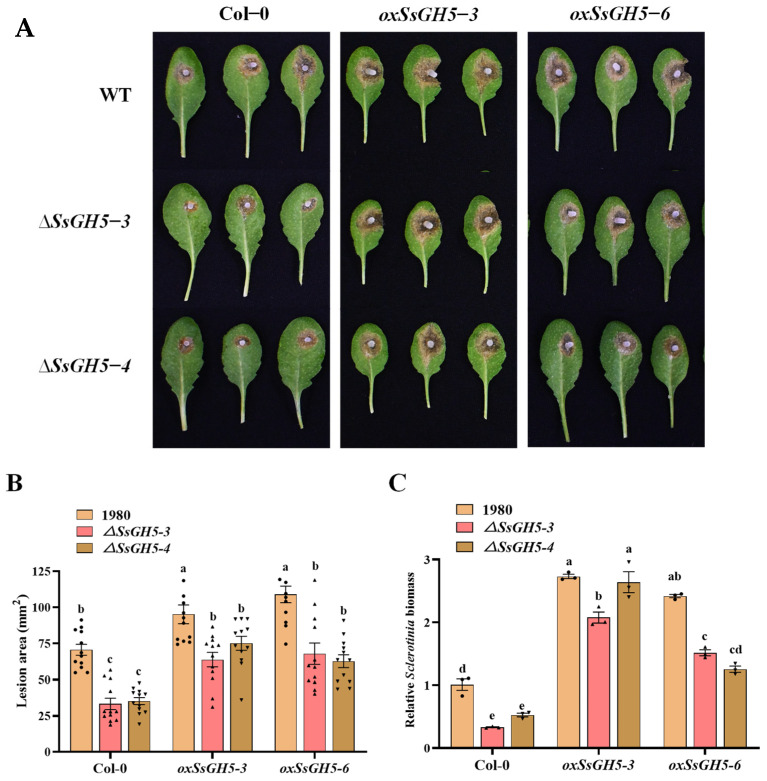
SsGH5 is an important pathogenic factor of *S. sclerotiorum* infection. (**A**) Virulence assay of the *S. sclerotiorum* WT and *ΔSsGH5* mutants on Col-0 and *35S:SsGH5 Arabidopsis* lines at 36 hpi. (**B**) Measurement of lesion area by the cross-over method. (**C**) Equal area samples from infected sites were used to extract DNA, and the relative biomass was analyzed by RT-qPCR. These values are the mean ± S.E. Different letters on the same graph indicate statistical significance at *p* < 0.01 using one-way ANOVA.

To further confirm that SsGH5 plays an important role during *S. sclerotiorum* infection and is involved in the interaction with plants, we inoculated the WT, *ΔSsGH5-3* and *ΔSsGH5-4* onto the leaves of Col-0 and *SsGH5* transgenic lines (*oxSsGH5-3* and *oxSsGH5-6*) for virulence assays. Statistical results showed that *ΔSsGH5-3* and *ΔSsGH5-4* resulted in a significantly lower lesion area on Col-0 compared to the WT strain, but the *ΔSsGH5* knockout mutants appeared to show a significant recovery of virulence on *SsGH5* transgenic lines, and the lesion area caused by them recovered to the level of the WT strain infesting Col-0 (Figure 5A,B). And the biomass statistics also show that the *Sclerotinia* biomass of *ΔSsGH5* knockout mutants significantly recovered after inoculating onto *SsGH5* transgenic lines (Figure 5C). The above results demonstrate that SsGH5 is important for the virulence of *S. sclerotiorum* and is likely involved in interaction between *S. sclerotiorum* and host plants.

## 3. Discussion 

Glycosyl hydrolase 5 family proteins have been studied for decades and are involved in many biomolecular hydrolysis processes, it has been found that the substrates of these enzymes are xylan, xyloglucan, dextran, cellulose and hemicellulose [35,37,38]. In the present study, we sought to investigate the function of SsGH5 in *S. sclerotiorum*. We found that SsGH5 is a secreted protein that is highly conserved in *Ascomycetes* (Figure 1). *SsGH5* deletion mutants showed a reduced growth rate on medium with β-glucan as the sole carbon source, suggesting that SsGH5 functions as a glucanase. (Figure 2E). CWDEs are generally induced to be expressed by the host during the infection stage of plant pathogens, and the transcriptomic data also suggested that SsGH5 might be significantly induced as a CWDE of *S. sclerotiorum* during the infection stage [8,9]. RT-qPCR results further comfirmed that SsGH5 gene expression was indeed induced during *S. sclerotiorum* infection of *Arabidopsis* (Figure 2A). This further suggests that SsGH5 is a CWDE associated with pathogenesis in *S. sclerotiorum*.

Numerous studies have shown that CWDEs are secreted by *S. sclerotiorum* to degrade plant cell walls for nutrients [21,39]. *S. sclerotiorum* produces several forms of pectinases, such as polygalacturonases (PGs) to degrade pectin, which are important for the pathogenicity of this fungus [40,41]. In addition, GH proteins predominate in CWDEs and are secreted by necrotrophic phytopathogenic fungi that are essential in degrading the cell wall and facilitating the pathogen’s full virulence [27,42,43]. In this study, we reported that SsGH5 was closely associated with the virulence of *S. sclerotiorum*. SsGH5 is not required for normal growth and does not affect oxalic acid production (Figure 2B–D), but SsGH5 knockout mutants showed significantly reduced virulence on both *B. napus* and *Arabidopsis* leaves (Figure 3A,D), indicating that this gene significantly affects the virulence of *S. sclerotiorum*. The importance of GH proteins for the virulence of plant pathogens is likely to be dependent on their hydrolase activity. Correspondingly, the glucanase activity of SsGH5 deletion mutants was decreased. It is noteworthy that the reduction in hydrolase activity observed in SsGH5 deletion mutants does not align consistently with the decrease in virulence. These results may be due to the existence of redundant genes in *S. sclerotiorum*, and SsGH5 may also have other functions. In fact, previous studies have shown that GH proteins were also involved in plant–pathogen interaction. For instance, Ebg1, as a glucanase in *M. oryzae*, can hydrolyze its own glucan, thereby achieving immune escape [33]. Interestingly, plants seem to have developed receptors capable of recognizing the GH family of proteins. Ebg1 also can be recognized by plants to trigger immune responses and cause cell death [33]. This is not an isolated example, XEG1 is important for virulence and can be recognized by plant RXEG1 as a PAMP and trigger intense cell death [29,30,44]. PpOPEL and VdXyn4 containing the GH domains could cause plant cell death, and plants pretreated with these proteins exhibit robust induced resistance [26,45]. These studies showed that the roles of GH in the pathogenesis are diverse and may be more complicated than we thought. Notably, we obtained transgenic *Arabidopsis* that stably expressed SsGH5 and did not observe cell death in SsGH5 transgenic plants (Figure S2). However, both the chitin-triggered ROS burst and the activation of MAPKs were significantly suppressed in the SsGH5 transgenic lines (Figure 4G,H), which supported the PTI of *Arabidopsis* was inhibited by SsGH5. As far as we know, this is the first report that GH protein of plant pathogen has the function of inhibiting the immunity of host plants. Most importantly, we found that SsGH5 transgenic *Arabidopsis* was significantly susceptibility to necrotrophic fungal pathogens including *S. sclerotiorum* and *B. cinerea* (Figure 4A–F). Furthermore, expression of SsGH5 in *Arabidopsis* plants restored the loss of virulence of the *ΔSsGH5* mutants (Figure 5). The results further indicated that the SsGH5 could play important roles in plants during the pathogenic process of *S. sclerotiorum*.

Taken together, our results indicate that *SsGH5* is not only essential for *S. sclerotiorum* virulence, but also has a significant effect on plant immunity, which are different from previous research in which GH family proteins caused plant cell death and activated plant immune responses. Moreover, the subcellular localization of SsGH5 suggests that it may interact with plant immunity-related proteins or receptors in the extracellular space to inhibit plant immunity and promote the colonization of *S. sclerotiorum*. The specific target of SsGH5 and the biological function and mechanism of its interaction need to be further studied.

## 4. Methods

### 4.1. Fungal Strains, Plant Materials and Growth Condition Conditions

The *S. sclerotiorum* wild-type strain 1980 (ATCC 18683) was obtained from diseased bean plants in Scottsbluff, NE [46]. And the WT strain was cultured on potato dextrose agar (PDA) (Lot 3199948, Becton Dickinson and Company, Franklin Lakes, NJ, USA) plates in a dark greenhouse at a constant temperature of 20 °C. The gene knockout mutants were cultured on PDA plates amended with hygromycin B. 100 μg·mL^−1^ (Sigma, St. Louis, MO, USA). The above PDA plates were placed in a 4 °C refrigerator (MPR-512HI, Alphavita, Dalian, China) for long-term storage.

All *Arabidopsis* plants used in this study were in the Columbia-0 (Col-0) genetic background. *Arabidopsis* lines were grown in growth chamber soil at 22 °C, 75 mE^−2^·s^−1^ (T5 LED tube light, 4000 K). The light/dark photoperiod was 12 h and the relative humidity was 40~60%.

### 4.2. Bioinformatics Analysis

The SsGH5 protein sequence (*Ss1G_00746*) was downloaded from the NCBI GenBank database. SIGNALP 4.0 and SIGNALP 4.1 were used for signal peptide prediction [47,48], and DeepTMHMM (https://dtu.biolib.com/DeepTMHMM, accessed on 15 June 2022) was used for prediction of trans-membrane helices. Phylogenetic analysis was performed to reconstruct the phylogenetic tree with MEGA X using the maximum likelihood method.

### 4.3. Plasmid Construction and Generation of Transgenic Plants

The coding sequence of SsGH5 was amplified from *S. sclerotiorum* cDNA with primers containing homologous fragments of the pCNF3 vector. The fragment was then cloned into CaMV 35S promoter-driven binary expression vectors pCNF3 with 3 × flag and pTF101 with GFP tags fused at the C terminus for the SsGH5-GFP localization observation in *N. benthamiana* and *Agrobacterium*-mediated floral dipping of *Arabidopsis*. SsGH5 and SsGH5^sp^ subcloned into the pSUC_2_ vector for expression in the YTK12 yeast strain to verify secretion using the same methods via homologous recombination (Figure S1a) (Vazyme, Cat. C113-02).

*Agrobacterium tumefaciens* GV3101 carrying pCNF3-SsGH5−3 × flag was cultured overnight in LB liquid medium containing 50 mg·mL^−1^ rifampicin and 50 mg·mL^−1^ kanamycin. After centrifugation at 4000 rpm for 5 min, the bacteria were suspended in *Agrobacterium* infiltration buffer containing 5% sucrose and 0.04% (*v*/*v*) silwetL-77 at an optical density (OD) 600 = 0.8. *Arabidopsis* buds were thoroughly immersed in the bacterial suspension, and plants were moisturized for 8 h after immersion. Plants were then maintained at 22 °C and 45% relative humidity with a 16 h light/8 h dark photoperiod, conditions for seed harvesting.

Transgenic *Arabidopsis* was screened with 1/2 MS (Lot P17261.01, Duchefa Biochemie, Haarlem, The Netherland) containing 50 mg·mL^−1^ kanamycin and further confirmed by immunoblotting using anti-flag antibody (F1804, Sigma, St. Louis, MO, USA).

### 4.4. Gene Knockout and Complementation of S. sclerotiorum

Obtaining gene knockout mutants of the *SsGH5* gene from *S. sclerotiorum* was accomplished by the split-marker technique [49]. The knockout strategy of this technique is illustrated in Supplementary Figure (Figure S1b). Two fragments of approximately 1000 bp, *SsGH5*-5′ and *SsGH5*-3′, were amplified from genomic DNA by PCR reactions with primers P1/P2 (both containing *Sal* I site) and P3/P4 (both containing *Xba* I site) using KOD DNA Polymerase (TOYOBO KMM-101, Japan) (Table S1). These products were cloned into the *Sal* I and *Xba* I sites in the pUCH18 vector containing the hygromycin-resistant cassette, respectively [18]. The pUCH18-*SsGH5*-5′ and pUCH18-*SsGH5*-3′ were obtained after successful cloning. The fusion sequences *SsGH5*-5′ and *SsGH5*-3′ with two truncated hygromycin-resistant genes, *SsGH5*-5′-HY and YG-*SsGH5*-3′, were mass amplified with primers P1/HY and YG/P4, respectively (Table S1). After the amount of purified *SsGH5*-5′-HY and YG-*SsGH5*-3′ DNA fragments both reached 10 μg or more, they were mixed in equimolar amounts and used for transformation.

For preparation and transformation of *S. sclerotiorum* protoplasts, the methodology of the previous study was adopted [18]. Mycelial balls grown in PDB for 36 h were lysed with lysing enzymes from *Trichoderma harzianum* (Cat. L1412, Sigma, St. Louis, MO, USA) to obtain fresh protoplasts. Fragments of *SsGH5*-5′-HY and YG-*SsGH5*-3′ were transferred into the protoplasts of WT strains covered with RM (0.7 M Sucrose, 0.5 g·L^−1^ Yeast Extract, 10 g·L^−1^ Agar) medium containing 200 μg·mL^−1^ hygromycin B. Putative transformants with hygromycin B resistance were obtained after 5 to 7 days. After verification of correct substitution of site according to the knockout strategy schematic, they were induced to produce sexual state ascospores, which were screened for hygromycin resistance in order to obtain pure syngeneic knockout mutant strains. The pure knockout mutant strains were validated by PCR and RT-PCR (Figure S1c,d).

### 4.5. Determination of the Biological Characteristics of S. sclerotiorum Transformants

Transformant strains of *S. sclerotiorum* (*ΔSsGH5* mutant and WT strains) were characterized for growth rate, colony morphology, acid production capacity and virulence. The WT and knockout strains were inoculated in the center of PDA plates for 3 days at 20 °C to measure the growth rate and incubated continuously until 14 days to record their colony morphology by photograph. To assay the ability of oxalic acid production, the wild-type strain and deletion mutant strains were inoculated into the center of PDA plates containing 0.005% (*w*/*v*) bromophenol blue dye and grown at 20 °C for 36 h, and their acid production ability was qualitatively characterized by vitual observation. The WT strain and the knockout strains were inoculated into the center of YGA plates and incubated at 20 °C for 3 days, and the growth rate was measured and the colony morphology was recorded by photographs (YGA medium: 0.5% (*w*/*v*) yeast extract, 1% (*w*/*v*) β-glucan (Aladdin SKU G304913) and 1% (*w*/*v*) agar).

### 4.6. The Fungal Inoculation Assay

For the fungal inoculation assay, agar discs (2 mm diameter) were punched from the actively growing edge of the fungal 2 × SY plates (SY medium: 0.5% (*w*/*v*) sucrose and yeast extract, 1% (*w*/*v*) agar) and inoculated on leaves of 4–5-week-old *Arabidopsis* plants and incubated at 22 °C. Eight to twelve biological replicates per treatment were taken from eight *Arabidopsis* plants. At the same time, the true leaves of *B. napus* grown for 30~40 days were selected for inoculation with fungi for virulence determination. Fungal virulence was assessed in two ways: macroscopically by measuring the long and short axes of the lesion with calipers, and molecularly by measuring the ratio of *S. sclerotiorum* DNA to host plant DNA in infected leaves. Disease lesion area was calculated based on the elliptical area formula. Relative pathogen biomass was based on the ratio of pathogen DNA to host plant DNA, fixed-mass samples from all infected sites were selected to extract DNA and amplified by quantitative PCR of genomic DNA for the *S. sclerotiorum β-Tubulin* gene or *B. cinerea actin* gene, and the *A. thaliana UBQ5* gene or *B. napus actin* gene (Table S1 for primers) by quantitative PCR of genomic DNA. Three replicates (each replicate contained 2–4 diseased leaves) were set up for each treatment, and each experiment was performed three times.

### 4.7. ROS Production Analysis and the MAPK Activation Assay

The third or fourth pair of true leaves of 4-week-old soil-grown *Arabidopsis* plants were cut into leaf discs (5 mm in diameter) and further cut into leaf strips. The leaves were left to float in 96-well plates with 100 µL ddH_2_O and shaken gently overnight to eliminate the wounding effect. For the assay, ddH_2_O was replaced by 100 µL reaction solution containing 50 µM L-012 (CAS:143556-24-5, Wako, Japan), 10 µg·mL^−1^ horseradish peroxidase (Sigma Cat. P6782) and chitin. Measurements were performed immediately after the addition of the reaction solution on a Multimode Reader Platform (SPARK 10M, Tecan Austria GmbH, Männedorf, Switzerland) and the values generated by ROS represent the relative light units of different plants.

*Arabidopsis* seedlings grown on 1/2 MS plates for 10 days were transferred to 1 mL sterilized ddH_2_O, recovered overnight, and then treated with the indicated concentrations of chitin for 0, 5, 15 and 30 min. The total protein of the samples was extracted with protein extraction buffer (20-mM Tris-HCl, pH 7.5, 100-mM NaCl, 1-mM EDTA, 10% glycerol and 1% Triton X-100) and the samples were kept at 95 °C for 10 min. The supernatant was collected after centrifugation at 12,000 rpm for 2 min, and the protein samples containing 1 × SDS buffer were loaded onto 10% (*v*/*v*) SDS-PAGE gels and immunoblotted with anti-PERK1/2 antibody to detect pMPK3, pMPK4 and pMPK6 (Cat. 9101S, CST, Boston, MA, USA).

### 4.8. The Secretion Trap Screen Assay

In this study, the predicted signal peptide fragment of the SsGH5 gene was fused to the N-terminus of the secretion-defective convertase gene (suc2) in the vector pSUC2 and then transformed into the yeast strain YTK12. Candidate yeast transformants were screened and cultured on CMD-W medium (Coolaber, Beijing, China) and YPRAA (10 g·L^−1^ yeast extract, 20 g·L^−1^ peptone, 20 g·L^−1^ raffinose, 2 mg·L^−1^ antimycin A, and 2% agar) medium (Macklin, Shanghai, China), and strains with secretion activity were able to grow on YPRAA. TTC (Sigma-Aldrich CAS NO 298-96-4) was used to assay the secretion sucrase activity of the candidate yeast transformants. The candidate yeast transformants were incubated in 10% sucrose solution at 30 °C for 35 min, then the supernatant was centrifuged and the final concentration of 0.1% TTC reagent was added and left at room temperature for 5 min to observe the color change in the test tube. The positive reaction changed from colorless to dark red, and the Avr1b^sp^ transformant, YTK12-pUSC2 and YTK12 strains were used as positive and negative controls, respectively.

### 4.9. RNA Isolation, cDNA Synthesis and RT-qPCR Analysis

Fungal samples were ground to a powder in liquid nitrogen, total RNA was extracted using trizol, and DNA was removed using RNase-free recombinant DNase I (2270A, Takara, Beijing, China). To examine the expression patterns of the *SsGH5* and WT strains, mycelium was cultured on PDA for 36 h and inoculated onto *Arabidopsis* leaves. Mycelia were harvested at 0, 1, 3, 6, 9,12, 24 and 48 h to extract nucleic acids. The concentration of total RNA was quantified using a spectrophotometer (Thermo, Waltham, MA, USA), and first-strand cDNA was synthesized using Easy Script One-Step gDNA Removal and cDNA Synthesis SuperMix (AE311-02, Transgen, Beijing, China). RT-PCR was performed in a CFX96 RT-PCR Detection System and TransStart Green qPCR SuperMix (Transgen AQ101-01). RNA samples for each RT-PCR were normalized with the *β-tubulin* gene *Sstub1* of *S. sclerotiorum*. For the *SsGH5* gene, RT-PCR assays were repeated at least twice, with three biological replicates each time.

## 5. Conclusions

In summary, our study determined that SsGH5, a GH5-family-secreted protein, as significantly upregulated during infection of *S. sclerotiorum*. SsGH5 is indispensable for the full virulence of *S. sclerotiorum.* In addition, SsGH5 can suppress *Arabidopsis* immunity and cause *Arabidopsis* to be more susceptible to *S. sclerotiorum* and *B. cinerea*. These results showed that SsGH5 is likely to be involved in the interaction between *S. sclerotiorum* and the host plant. Further screening the target protein of SsGH5 and exploring the biological function and mechanism may reveal a new model for pathogen–plant interactions.

## Data Availability

The sequence of SsGH5 was deposited in the GenBank database, with accession numbers XP_001598657.1 (hypothetical protein SS1G_00746 [Sclerotinia sclerotiorum 1980 UF-70]—Protein—NCBI (nih.gov)).

## Supplementary Materials

The following supporting information can be downloaded at: https://www.mdpi.com/article/10.3390/ijms25052693/s1.
