# Supplementary material for: A Glycosyl Hydrolase 5 Family Protein Is Essential for Virulence of Necrotrophic Fungi and Can Suppress Plant Immunity
Source: Int J Mol Sci. 2024 Feb 26;25(5):2693. doi: 10.3390/ijms25052693 (PMC10932230; doi:10.3390/ijms25052693)
Supplement: Supplementary file 1 [file ijms-25-02693-s001.zip › ijms-2840815-supplementary.pdf]

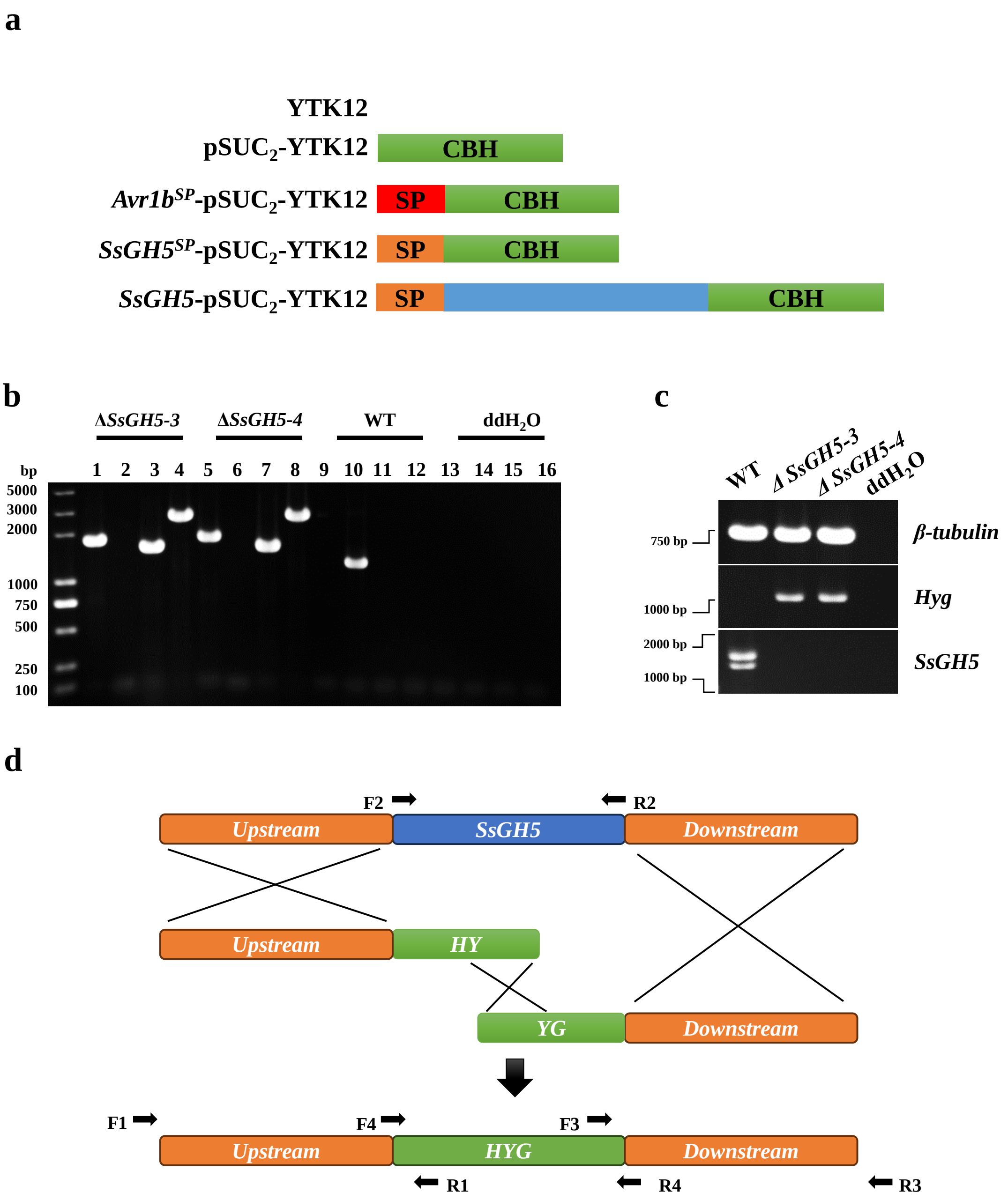

Figure S1. Generation and analysis of *ΔSsGH5* mutants. **a** The signal peptide of effector Avr1b, SsGH5 and the full-length amino acid sequence of SsGH5 were constructed to the pSUC2 vector, respectively, and subsequently transferred into the yeast strain of YTK12 and used to test for secretion. **b** PCR validation of knockout and complementary strains. The full length of the *HptII* fragment was amplified with primer F4/R4 (lane: 4, 8, 12, 16); upstream of the trpC promoter overlapping with SsGH5 was amplified with primer F1/R1. (lane: 1, 5, 9, 13); trpC terminator

overlapping with SsGH5 downstream, amplified with primer F3/R3 (lane: 3, 7, 11, 15); *SsGH5* full length amplified with primer F2/R2 (lane: 2, 6, 10, 14). Lane M, DL5000 marker. **c** Detection of mRNA expression levels of *ΔSsGH5* mutants and complementary mutants by reverse transcription PCR. **d** Schematic representation of targeted gene recombination substitutions, with different colors shown indicate *HptII* cassette (green), *SsGH5* (blue) and flanking sequences (orange). The scheme is not drawn to scale.

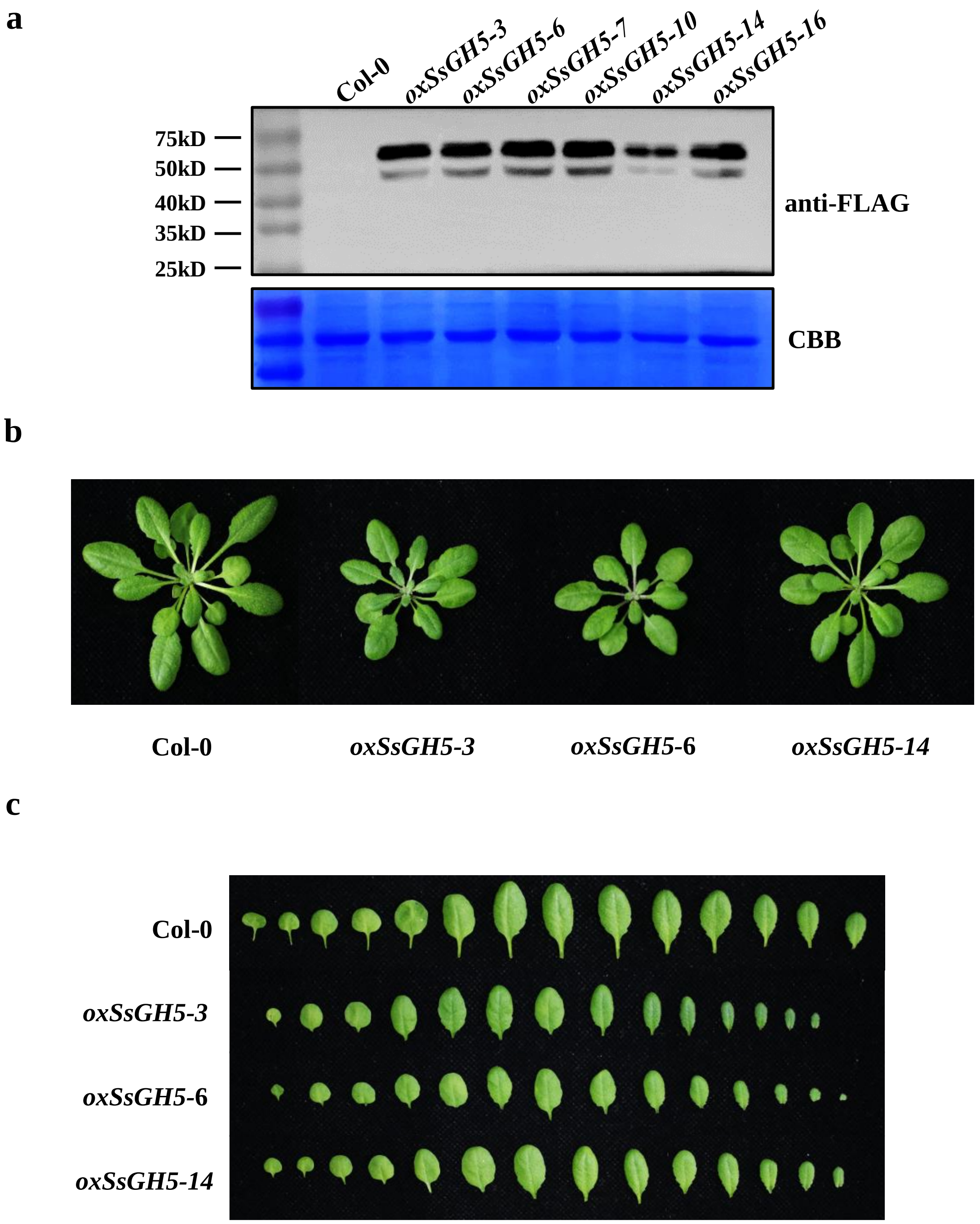

Figure S2. Confirmation of *SsGH5* transgenic plants. **a** Western blot detection of SsGH5 expression in transgenic *Arabidopsis* lines. Anti-FLAG M2 mAb was used for western blotting and stained with CBB as a loading control. **b**, **c** Morphological characteristics of *SsGH5* transgenic *Arabidopsis* lines.

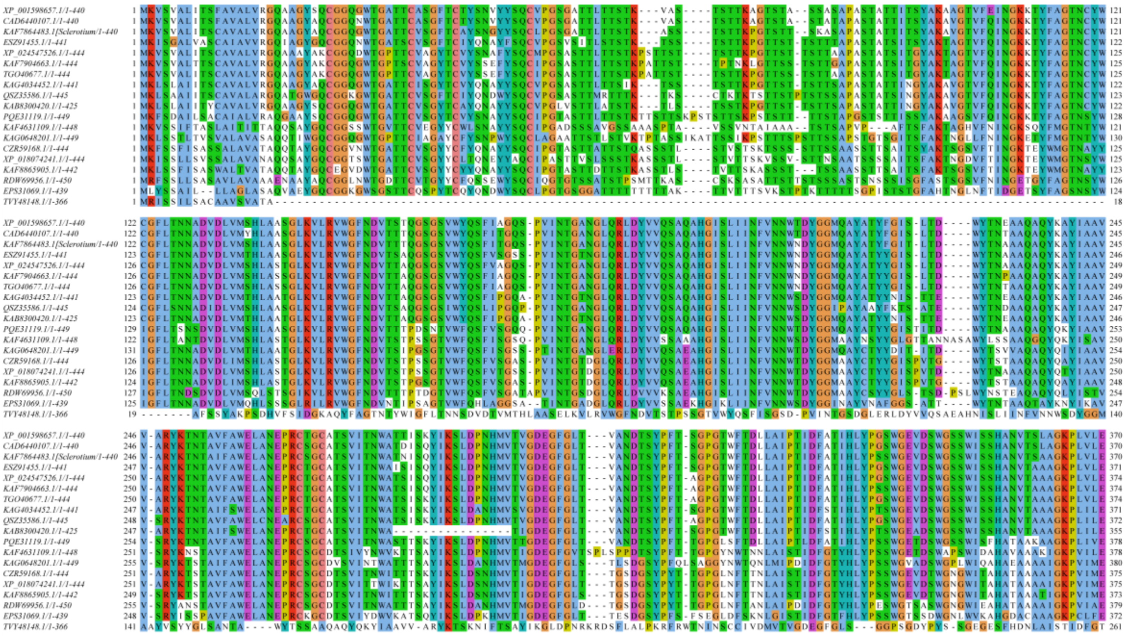

Figure S3. Sequence multiple alignment of SsGH5 homologs. Amino acid multiple comparisons illustrated that the amino acid sequence of SsGH5 is prevalent in different ascomycetous fungi and that both arginine and lysine show a high degree of conservation among the homologous proteins.

Supplementary Table S1. List of primers used in the study

**Primer name**	**Sequence 5'-3'**	**Purpose**
GH5-Sal1-F（P1）	GTCGAC GCGACGTATTCATCAGACATGACT	SsGH5 5' flanking sequence
GH5-Sal1-R（P2）	GTCGAC TCTGGAGATATTTGGCCAAACCT	SsGH5 5' flanking sequence
GH5-Xba1-F（P3）	TCTAGA GGAGGTGCAATTTTGCGCT	SsGH5 3' flanking sequence
GH5-Xba1-R（P4）	TCTAGA CATGCAGATAGAAGCTGACTCACG	SsGH5 3' flanking sequence
YG-F	GATGTAGGAGGGCGTGGATATGTCCT	Hyg Split 3' construct
HY-R	GTATTGACCGATTCCTTGCGGTCCGAA	Hyg Split 5' construct
GH5UYZ-F (F1)	TTGACTCATGGGGAGGTGCACG	SsGH5 5'upstream validation
GH5UYZ-R (R1)	CCTCGTTCCTGTCTGCTAATAAGAGTCAC	SsGH5 5'upstream validation
YZ-GH5-F (F2)	GGTATCAGTTGCGCTTATCACGTCC	SsGH5 PCR
YZ-GH5-R (R2)	AACGGCCTTTGCGTTCATAGCAG	SsGH5 PCR
GH5DYZ-F (F3)	CTACGAGACTGAGGAATCCGCTCTT	SsGH5 3'downstream validation
GH5DYZ-R (R3)	GAAGCTGACTCACGCCGACGA	SsGH5 3'downstream validation
YZ-HYG-F (F4)	AGAAGATGATATTGAAGGAGCACT	HYG PCR
YZ-HYG-R (R4)	AAGAAGGATTACCTCTAAACAAGTGT	HYG PCR
SsGH5-EcoR1-F	GAATTC ATGAAGGTATCAGTTGCGCTTA	Yeast secretion trap system
$SsGH5^{SP}$-EcoR1-R	GAATTC ACCTTGTCCTCCACACTGTGA	Yeast secretion trap system
SsGH5-EcoR1-R	GAATTC TGGAACGGCCTTTGCG	Yeast secretion trap system
SsGH5-F (recombination)	agaaggcctactagt ATGAAGGTATCAGTTGCGCTTA	Subcellular localization
SsGH5-R (recombination)	gacggatccactagt TGGAACGGCCTTTGCG	Subcellular localization
SsGH5-F (recombination)	cctactagtggatcc ATGAAGGTATCAGTTGCGCTTA	Transgenic *Arabidopsis thaliana*
SsGH5-R (recombination)	accgtcgacggatcc TGGAACGGCCTTTGCG	Transgenic *Arabidopsis thaliana*
SsTub-F	ATAACTTCGTTTTCGGTCAATCCG	RT-PCR
SsTub-R	ATAACTTCGTTTTCGGTCAATCCG	RT-PCR
HYGcds-F	ATGAAAAAGCCTGAACTCACCGCGA	RT-PCR
HYGcds-R	CTATTCCTTTGCCCTCGGACGAGTG	RT-PCR
SsGH5-F	ATGAAGGTATCAGTTGCGCTTA	RT-PCR
SsGH5-R	TGGAACGGCCTTTGCG	RT-PCR
SsTub-qF	CTGTATCGACAACGAGGCTCTCT	RT-qPCR
SsTub-qR	GAGTTAAGTTGACCAGGGAAACG	RT-qPCR
SsGH5-qF	CGAGTCTGGGGCTTCAATGAC	RT-qPCR
SsGH5-qR	GATGAGACTGATTCCGTGAGC	RT-qPCR
SsTub-F	ACCTCCATCCAAGAACTC	Biomass determination
SsTub-R	GAACTCCATCTCGTCCAT	
AtUBQ5-F	ACACCAAGCCGAAGAAGA	Biomass determination
AtUBQ5-R	TCCACAGGTTGCGTTAGG	
BnActin-F	ATTGGTGTTGGGGTTGGG	Biomass determination
BnActin-R	TGGCTGTTTTGCGTATTCAT	
BcActin-F	CTTCGTGTAGCACCAGAGGAG	Biomass determination
BcActin-R	GAGAGGACGGCTTGAATAGAGA	
